# Supplementary material for: Early prediction of disease progression in COVID-19 pneumonia patients with chest CT and clinical characteristics
Source: Nat Commun. 2020 Oct 2;11:4968. doi: 10.1038/s41467-020-18786-x (PMC7532528; doi:10.1038/s41467-020-18786-x)
Supplement: Supplementary file 1 — Supplementary Information [file 41467_2020_18786_MOESM1_ESM.pdf]

Supplementary Information

Early prediction of disease progression in COVID-19 pneumonia patients with chest CT and clinical characteristics

Feng et al.

Supplementary Table 1 Clinical and CT characteristics between patients with and without bacterial co-infection during hospitalization in the derivation cohort.

Variables	Without (n = 124)	With (n= 17)	P value
Age (years)	41 (34-53)	56 (38-66)	0.031
Male gender	62 (50.0%)	10 (58.8%)	0.495
Exposure history in Wuhan within 2 weeks			0.664
Yes	66 (53.2%)	10 (58.8%)	
No	58 (46.8%)	7 (41.2%)	
Smoking history	7 (5.7%)	0 (0)	0.315
Comorbidities			
Any	24 (19.4%)	9 (52.9%)	0.002
Diabetes	7 (5.7%)	1 (5.9%)	0.968
Hypertension	15 (12.1%)	6 (35.3%)	0.012
Cardiovascular disease	2 (1.6%)	1 (5.9%)	0.322
COPD	2 (1.6%)	2 (11.8%)	0.071
Cerebrovascular disease	1 (0.8%)	0 (0)	0.710
Hepatitis B infection	3 (2.4%)	1 (5.9%)	0.405
Signs and symptoms			
Fever	90 (72.6%)	15 (88.2%)	0.165
Cough	68 (54.8%)	6 (35.3%)	0.130
Sputum production	13 (10.5%)	3 (17.7%)	0.383
Fatigue or myalgia	25 (20.2%)	6 (35.3%)	0.158
Anorexia	4 (3.2%)	1 (5.9%)	0.479
Diarrhea	4 (3.2%)	2 (11.8%)	0.154
Shortness of breath	3 (2.4%)	2 (11.8%)	0.110
Percutaneous oxygen saturation (%)	97.4 (95.8-98.4)	98.1 (95.9-99.0)	0.231
Laboratory findings			
Platelet count (×10⁹/L)	168.0 (137.0-226.0)	148.0 (119.5-190.0)	0.105
White blood cell count (×10⁹/L)	4.4 (3.3-5.5)	4.6 (3.5-5.0)	0.858
Neutrophil count (×10⁹/L)	2.8 (2.1-3.7)	3.1 (2.0-3.8)	0.707
Lymphocyte count (×10⁹/L)	1.1 (0.8-1.4)	0.9 (0.7-1.2)	0.113
NLR	2.5 (1.9-3.7)	3.2 (2.3-5.1)	0.065
Alanine aminotransferase (U/L)	19.6 (14.5-28.4)	20.6 (15.2-32.2)	0.740
Aspartate aminotransferase (U/L)	23.4 (19.4-30.3)	27.4 (21.2-36.7)	0.064
Total bilirubin (μmol/L)	10.9 (8.3-15.3)	11.0 (7.9-16.1)	0.939
Albumin (g/L)	36.9 (34.7-40.1)	37.3 (34.6-40.9)	0.753
Creatinine (μmol/L)	50.4 (39.8-59.7)	49.9 (40.8-62.4)	0.719
Creatine kinase (U/L)	69.6 (40.0-125.7)	90.1 (55.4-138.6)	0.167
Lactic dehydrogenase (U/L)	171.6 (134.2-211.9)	195.5 (150.6-252.7)	0.097
C-reactive protein (mg/L)	17.2 (6.9-35.9)	18.7 (10.7-35.4)	0.427
CT features			
Number of lobes involved			0.492
One lobe	12 (9.7%)	2 (11.8%)	
Two lobes	23 (18.6%)	2 (11.8%)	

Three lobes	15 (12.1%)	0 (0)	
Four lobes	24 (19.3%)	5 (29.4%)	
Five lobes	50 (40.3%)	8 (47.0%)	
Number of segments involved	9 (5-12)	11 (5-14)	0.257
Bilateral involvement	109 (87.9%)	14 (82.4%)	0.520
Distribution pattern			0.817
Peripheral	63 (50.8%)	8 (47.1%)	
Central	2 (1.6%)	0 (0)	
Mixed	59 (47.6%)	9 (52.9%)	
GGO	118 (95.2%)	17 (100%)	0.354
Consolidation	109 (87.9%)	11 (64.7%)	0.012
GGO with consolidation	102 (82.3%)	11 (64.7%)	0.089
Crazy-paving	36 (29.0%)	6 (35.3%)	0.597
Air bronchogram	73 (58.9%)	9 (52.9%)	0.642
Discrete nodules	9 (7.3%)	2 (11.8%)	0.516
Lymphadenopathy	6 (4.8%)	0 (0)	0.354
Pleural effusion	4 (3.2%)	0 (0)	0.452
CT severity score	6 (3-10)	7 (4-10)	0.817
Hospital length of stay (days)	20 (16-27)	35 (24-42)	< 0.001
Duration of viral shedding after illness onset (days)	14 (10-21)	32 (19-36)	< 0.001

Data are presented as median (IQR) or n (percentage). Differences between groups are analyzed using Student's t-test or Mann-Whitney U test for continuous variables and Chi-square test or Fisher's exact test for categorical variables. Two-sided P values are reported.

Abbreviations: COPD, chronic obstructive pulmonary disease; CT, computed tomography; GGO, ground-glass opacities; IQR, interquartile range; NLR, neutrophil-to-lymphocyte ratio.

Supplementary Table 2 Clinical and CT characteristics of patients with COVID-19 in the derivation cohort according to exposure history in Wuhan within 2 weeks.

Variables	Yes (n=76)	No (n=65)	P value
Age (years)	41 (35-52)	47 (31-57)	0.480
Hypertension	13 (17.1%)	8 (12.3%)	0.425
Lymphocyte count ($\times 10^9$/L)	1.0 (0.8-1.4)	1.1 (0.9-1.5)	0.258
NLR	2.6 (1.9-4.1)	2.5 (1.8-3.6)	0.562
Aspartate aminotransferase (U/L)	24.7 (19.5-30.5)	23.3 (20.2-31.4)	0.828
Albumin (g/L)	37.1 (34.5-40.0)	37.1 (35.0-40.3)	0.745
Lactic dehydrogenase (U/L)	174.0 (133.8-209.4)	182.0 (142.0-221.1)	0.326
C-reactive protein (mg/L)	19.0 (7.2-30.6)	16.2 (7.4-38.8)	0.864
Number of lobes involved	4 (2-5)	4 (2-5)	0.799
Number of segments involved	9 (4-12)	9 (5-13)	0.383
Crazy-paving	22 (29.0%)	20 (30.8%)	0.814
CT severity score	6 (4-9)	7 (3-10)	0.715

Data are presented as median (IQR) or n (percentage). Differences between groups are analyzed using Student's t-test or Mann-Whitney U test for continuous variables and Chi-square test or Fisher's exact test for categorical variables. Two-sided P values are reported.

Abbreviations: COVID-19, coronavirus disease 2019; CT, computed tomography; IQR, interquartile range; NLR, neutrophil-to-lymphocyte ratio.

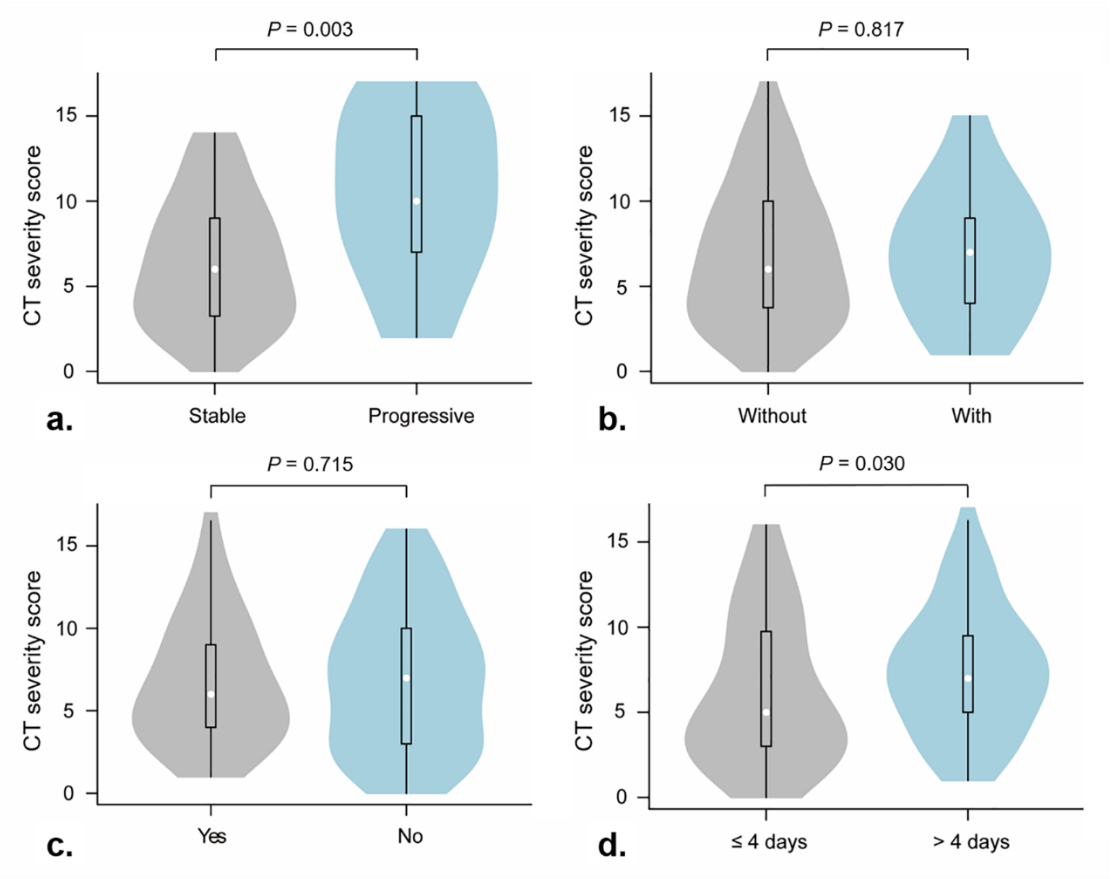

Supplementary Fig. 1: Comparison of CT severity scores between different groups. The violin plots of the CT severity score between stable and progressive groups (a), between patients with and without bacterial co-infection during hospitalization (b), between patients with and without exposure history in Wuhan within 2 weeks before illness onset (c), and between the patients who were admitted ≤ 4 days and > 4 days from symptom onset (d). The violins show a kernel density estimation of the groups. The white dots indicate the median, the black boxes indicate the IQR between the 25th and 75th percentile, and the thin black lines indicate the upper and lower adjacent values. Differences between groups are analyzed using Student's t-test or Mann-Whitney U test and two-sided P values are reported.

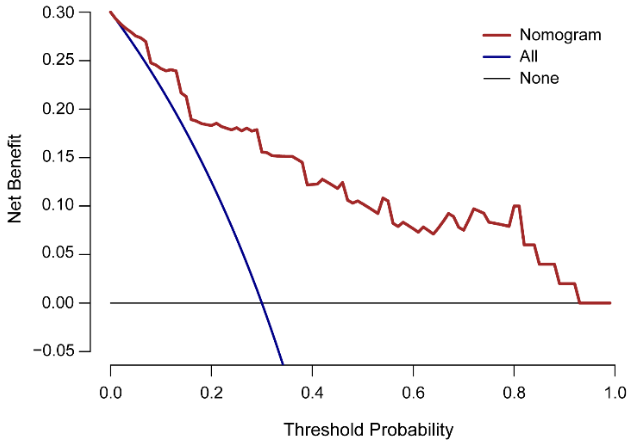

Supplementary Fig. 2: Decision curve analysis of the nomogram.

The red line represents the nomogram, and the blue and black lines represent the hypothesis that all patients had severe pneumonia and that no patients had severe pneumonia in the derivation cohort, respectively. The threshold probability is where the expected benefit of treatment is equal to the expected benefit of avoiding treatment. For example, if the possibility of severe pneumonia of a patient is over the threshold probability, then the treatment strategy for severe pneumonia should be adopted.
