# Supplementary material for: Early prediction of disease progression in COVID-19 pneumonia patients with chest CT and clinical characteristics
Source: Nat Commun. 2020 Oct 2;11:4968. doi: 10.1038/s41467-020-18786-x (PMC7532528; doi:10.1038/s41467-020-18786-x)
Supplement: Supplementary file 3 — Description of Additional Supplementary Files [file 41467_2020_18786_MOESM3_ESM.pdf]

Description of Additional Supplementary Files

File Name: Supplementary Data 1
Description: Chest CT and clinical characteristics of patients
