# Supplementary material for: Early prediction of disease progression in COVID-19 pneumonia patients with chest CT and clinical characteristics
Source: Nat Commun. 2020 Oct 2;11:4968. doi: 10.1038/s41467-020-18786-x (PMC7532528; doi:10.1038/s41467-020-18786-x)
Supplement: Supplementary file 5 — Reporting Summary [file 41467_2020_18786_MOESM5_ESM.pdf]

nature research

Corresponding author(s): Pengfei Rong, Wei Wang

Last updated by author(s): Sep 4, 2020

Reporting Summary

Nature Research wishes to improve the reproducibility of the work that we publish. This form provides structure for consistency and transparency in reporting. For further information on Nature Research policies, see Authors & Referees and the Editorial Policy Checklist .

Statistics

For all statistical analyses, confirm that the following items are present in the figure legend, table legend, main text, or Methods section.

n/a	Confirmed	
☐	☒	The exact sample size (*n*) for each experimental group/condition, given as a discrete number and unit of measurement
☐	☒	A statement on whether measurements were taken from distinct samples or whether the same sample was measured repeatedly
☐	☒	The statistical test(s) used AND whether they are one- or two-sided<br>*Only common tests should be described solely by name; describe more complex techniques in the Methods section.*
☐	☒	A description of all covariates tested
☐	☒	A description of any assumptions or corrections, such as tests of normality and adjustment for multiple comparisons
☐	☒	A full description of the statistical parameters including central tendency (e.g. means) or other basic estimates (e.g. regression coefficient) AND variation (e.g. standard deviation) or associated estimates of uncertainty (e.g. confidence intervals)
☐	☒	For null hypothesis testing, the test statistic (e.g. *F*, *t*, *r*) with confidence intervals, effect sizes, degrees of freedom and *P* value noted<br>*Give P values as exact values whenever suitable.*
☒	☐	For Bayesian analysis, information on the choice of priors and Markov chain Monte Carlo settings
☒	☐	For hierarchical and complex designs, identification of the appropriate level for tests and full reporting of outcomes
☐	☒	Estimates of effect sizes (e.g. Cohen's *d*, Pearson's *r*), indicating how they were calculated

Our web collection on statistics for biologists contains articles on many of the points above.

Software and code

Policy information about availability of computer code

Data collection	Microsoft Excel 2019 for clinical and imaging data collection.
Data analysis	Statistical analysis: IBM SPSS statistics software (version 22.0) and R software (version 3.6.1); Details of data analyses were described in the Methods section.

For manuscripts utilizing custom algorithms or software that are central to the research but not yet described in published literature, software must be made available to editors/reviewers. We strongly encourage code deposition in a community repository (e.g. GitHub). See the Nature Research guidelines for submitting code & software for further information.

Data

Policy information about availability of data

All manuscripts must include a data availability statement. This statement should provide the following information, where applicable:

- Accession codes, unique identifiers, or web links for publicly available datasets
- A list of figures that have associated raw data
- A description of any restrictions on data availability

The data supporting the main findings of this study are available from the corresponding authors upon reasonable request. A portion of data in this study is available within the Supplementary Information.

Field-specific reporting

Please select the one below that is the best fit for your research. If you are not sure, read the appropriate sections before making your selection.

Life sciences study design

All studies must disclose on these points even when the disclosure is negative.

Sample size	No statistical method was used to predetermine sample size. Consecutive patients with COVID-19 infection confirmed by virus nucleic acid testing and available chest CT scan on admission between January 17, 2020 and February 1, 2020 in five hospitals were screened, and some patients were excluded for the design of this study. The sample size was sufficient to demonstrate statistically significant differences in comparisons between groups by two-tailed Student t-test or Mann–Whitney U test.
Data exclusions	17% patients were excluded according to the pre-established exclusion criteria: 11) negative CT findings or severe COVID-19 on admission; 2) age younger than 18 years old.
Replication	The study included a sufficient human sample size, taking into account the expected variability. A nomogram based on multivariate logistic regression model in the derivation cohort was established, and external validation was performed with an independent cohort collected during the same period.
Randomization	Not relevant. Patients were selected and grouped based on the primary disease and progression of disease during observation.
Blinding	Baseline clinical data collection and CT images evaluation were performed in a blinding manner (blinded to the endpoint). Computational analysis was not performed blinded for the nature of supervised training or learning.

Reporting for specific materials, systems and methods

We require information from authors about some types of materials, experimental systems and methods used in many studies. Here, indicate whether each material, system or method listed is relevant to your study. If you are not sure if a list item applies to your research, read the appropriate section before selecting a response.

Materials & experimental systems

n/a	Involved in the study	
☒	☐	Antibodies
☒	☐	Eukaryotic cell lines
☒	☐	Palaeontology
☒	☐	Animals and other organisms
☐	☒	Human research participants
☒	☐	Clinical data

Methods

n/a	Involved in the study	
☒	☐	ChIP-seq
☒	☐	Flow cytometry
☒	☐	MRI-based neuroimaging

Human research participants

Policy information about studies involving human research participants

Population characteristics	We included 247 patients with COVID-19 infection and chest CT scan on admission, consisting of 126 males and 121 females, with a median age of 44 years and 46 years in the derivation and validation cohorts, respectively. The baseline demographic, epidemiological, laboratory, imaging characteristics, and clinical outcomes of patients in the present study are shown in Table 1.
Recruitment	Clinical and imaging data were obtained from the health records of Third Xiangya Hospital, Changsha Public Health Treatment Center, Second People's Hospital of Hunan, First Hospital of Yueyang, and Central Hospital of Shaoyang. Potential patients were screened based on the primary disease and progression of disease during observation. There is no self-selection bias.
Ethics oversight	The Institutional Review Board of Third Xiangya Hospital approved our study and informed consent was waived for the retrospective nature.

Note that full information on the approval of the study protocol must also be provided in the manuscript.
